# Group C Betacoronavirus in Bat Guano Fertilizer, Thailand

**DOI:** 10.3201/eid1908.130119

**Published:** 2013-08

**Authors:** Supaporn Wacharapluesadee, Chirapol Sintunawa, Thongchai Kaewpom, Kritsada Khongnomnan, Kevin J. Olival, Jonathan H. Epstein, Apaporn Rodpan, Paiboon Sangsri, Nirun Intarut, Ariya Chindamporn, Kanyarat Suksawa, Thiravat Hemachudha

**Affiliations:** Chulalongkorn University and King Chulalongkorn Memorial Hospital, Bangkok, Thailand (S. Wacharapluesadee, T. Kaewpom, K. Khongnomnan, A. Rodpan, N. Intarut, A. Chindamporn, K. Suksawa, T. Hemachudha);; Mahidol University, Nakornpathom, Thailand (C. Sintunawa); EcoHealth Alliance, New York, New York, USA (K.J. Olival, J.H. Epstein);; Ministry of Natural Resources and Environment, Bangkok (P. Sangsri)

**Keywords:** coronavirus, betacoronavirus, bat guano fertilizer, insectivorous bat, Thailand, viruses, zoonoses

**To the Editor:** Bats play a critical role in the transmission and origin of zoonotic diseases, primarily viral zoonoses associated with high case-fatality rates, including those caused by Nipah virus (NiV) and severe acute respiratory syndrome (SARS)–like coronavirus (CoV) infections (*1*). Recently, the World Health Organization (WHO) reported 44 confirmed cases of human infection with Middle East respiratory syndrome CoV, resulting in 22 deaths. Full-genome and phylogenetic analyses of these Middle East respiratory syndrome CoVs have been published elsewhere (*2*). The identified viruses from 2 patients (previously referred to as England/Qatar/2012 and EMC/2012) are genetically related and belong to group C betacoronavirus, which is most related to CoVs from *Nycteris* bats in Ghana and *Pipistrellus* bats in Europe (*2*,*3*). In addition, bat CoVs HKU4 and HKU5 originated from *Tylonycteris pachypus* and *Pipistrellus abramus* bats, respectively, in the People’s Republic of China (*4*). Bats are also known to harbor and transmit nonviral zoonotic pathogens, including the fungal pathogen *Histoplasma capsulatum,* which causes histoplasmosis in humans (*5*).

Bat guano is sold for use as a fertilizer in several countries, including Thailand, Indonesia, Mexico, Cuba, and Jamaica. The practice of collecting and harvesting bat guano may pose a considerable health risk because guano miners have a high level of contact and potential exposure to bat-borne pathogens. To assess pathogens in bat guano, we examined bat guano from a cave in the Khao Chong Phran Non-hunting Area (KCP-NHA) in Ratchaburi Province, Thailand, where bat guano was sold as agricultural fertilizer, for the presence of NiV, CoV, and *H. capsulatum* fungi. Bats from 14 species in 7 families have been found roosting within this area. *Tadarida plicata* bats are the most abundant species (2,500,000 bats), and 3 other species of bats found at the site each had thousands of members: *Taphozous melanopogon*, *Taphozous theobaldi*, and *Hipposideros larvatus*.

A random sample of dry bat guano, ≈100 g, was collected in a sterile plastic bag weekly from the main cave at KCP-NHA from September 2006 through August 2007. The specimens were sent for analysis by express mail (at room temperature within 2–3 days) to the WHO Collaborating Centre for Research and Training in the Viral Zoonoses Laboratory at Chulalongkorn University. Samples were frozen immediately at –80°C until nucleic acids were extracted and PCR assays were run. A total of 52 collected bat guano specimens were examined in this study.

Two aliquots of feces from each weekly specimen (104 samples total) were screened for CoV, NiV, and *H. capsulatum* by PCR. RNA was extracted from 10 mg of fecal pellet by using the QIAamp Viral RNA Mini Kit (QIAGEN, Hilden, Germany). CoV RNA was detected by using nested reverse transcription PCR with the degenerated primers to amplify the RNA-dependent RNA polymerase (*RdRp*) gene (*6*). NiV RNA was detected by duplex nested reverse transcription PCR (*7*). To detect *H. capsulatum* and other fungi, we extracted genomic DNA directly from bat guano by using the silica-guanidine thiocyanate protocol, NucliSense Isolation Reagent (bioMérieux, Boxtel, the Netherlands), according to the manufacturer’s protocol. We tested for fungal ribosomal DNA (rDNA) in extracted total nucleic acid specimens by using the PCR protocol designed to amplify all rDNA from 4 major fungus phyla at the internal transcribed spacer 1 and 2 regions (*8*).

Four (3.8%) of 104 samples were positive for CoV. They were collected on September 2, 2006 (KCP9), October 26, 2006 (KCP12), November 14, 2006 (KCP15), and March 4, 2007 (KCP31). Three of the 4 positive CoV sequences (KCP9, KCP12, and KCP15) were identical at 152 nt of the *RdRp* region (ATCGTGCTATGCCTAATATGTGTAGGATTTTTGCATCTCTCATATTAGCTCGTAAACACAATACTTGTTGTAGTGTTTCAGACCGCTtTtATAGACTTGCaAACGAGTGTGCGCAAGTCTTGAGTGAGTATGTGCTATGTGGTGGTGGCTAT) and phylogenetically clustered with the group C betacoronavirus (Figure), with 76%, 80%, and 77% nt identity to bat CoV HKU4, bat CoV HKU5, and human CoV EMC and England1_CoV, respectively. The other CoV sequence (KCP31: ATCGTGCACTTCCCAATATGATACGCATGATTTCCGCCATGATTTTGGGATCAAAGCATGTTACTTGCTGTGACACATCTGATAAGTATTACCGTCTTTGTAATGAGCTtGCACAAGTTTTGACAGAGGTTGTTTATTCTAATGGTGGTTTC) showed 82% nt identity with bat CoV HKU8, an alphacoronavirus. Although we recognize that longer sequences or full genomes may alter the topology of the phylogeny slightly and give stronger branch support, we expect that the overall topology and placement of these CoVs would remain consistent. Samples from particular bat species could not be identified because bats of different species roost in this cave, and samples were pooled during collection for bat guano fertilizer. The detection of CoVs in bat guano from the KCP-NHA cave in Ratchaburi was consistent with the previous finding of alphacoronavirus from *Hipposideros armiger* bats from the same province in 2007, but those researchers tested fresh bat feces (*9*).

**Figure:**
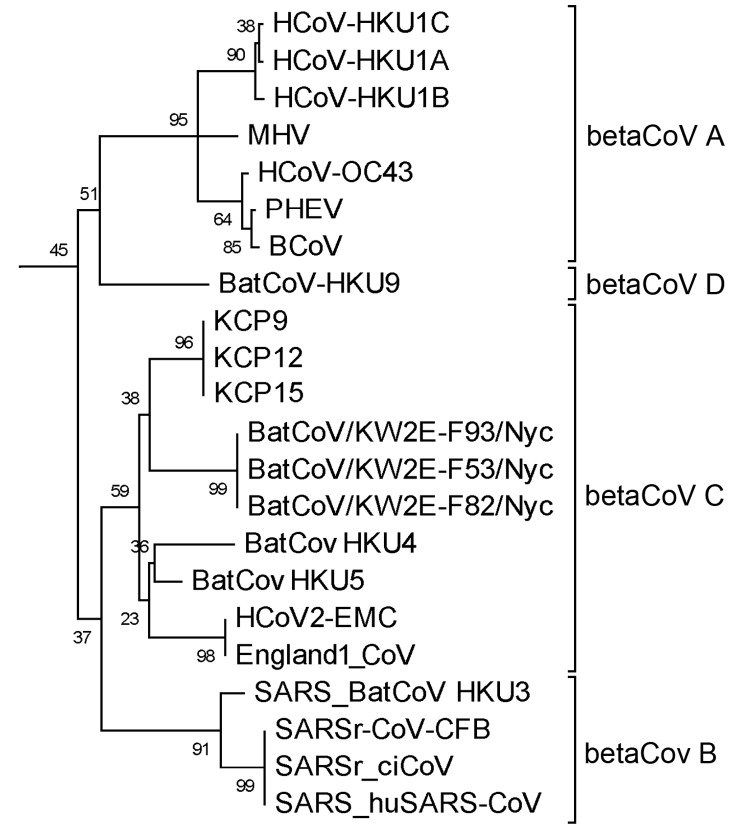
Phylogenetic tree of 4 coronaviruses (CoVs) isolated from bat guano collected in this study (KCP9, KCP12, KCP15, and KCP31); 35 additional human and animal CoVs from the National Center for Biotechnology Information database are included. Construction of the tree was based on 152 nt of the *RNA-dependent RNA polymerase* gene region by maximum-likelihood method and GTR+I model with the 1,000 bootstrap resampling method implemented in MEGA5 (http://megasoftware.net/). Numbers on branches indicate percentages of bootstrap support from 1,000 replicates. The scale bar indicates the estimated 0.1 nt substitutions per site. CoV genera (*Alphacoronavirus, Betacoronavirus, Gammacoronavirus, and Deltacoronavirus*) based on International Committee on Taxonomy of Viruses recommendation are indicated (alphaCoV, betaCoV, gammaCoV, and deltaCoV, respectively). BatCoV_HKU8, *Miniopterus* bat CoV HKU8 (EU420139); HCoV-NL63, human CoV NL63 (JX524171); PEDV, porcine epidemic diarrhea virus (NC_003436); BatCoV 512, *Scotophilus* bat CoV 512 (DQ648858); HCoV-229E, human CoV 229E (NC_002645); BatCoV-HKU2, *Rhinolophus* bat CoV HKU2 (EF203064); TGEV, transmissible gastroenteritis virus (AJ271965); FIPV, feline CoV (DQ010921); HCoV-HKU1C, human CoV HKU1C (DQ415913); HCoV-HKU1A, human CoV HKU1A (DQ415903); HCoV-HKU1B, human CoV HKU1B (AY884001); MHV, murine hepatitis virus (NC001846); HCoV-OC43, human CoV OC43 (AY585229); PHEV, porcine hemagglutinating encephalomyelitis virus (DQ011855); BCoV, bovine CoV (AF391541); BatCoV-HKU9, *Rousettus* bat CoV HKU9 (NC009021); BatCoV/KW2E-F93/Nyc, *Nycteris* bat CoV (JX899383); BatCoV/KW2E-F53/Nyc, *Nycteris* bat CoV (JX899384); BatCoV/KW2E-F82/Nyc, *Nycteris* bat CoV (JX899382); BatCoV HKU4, *Tylonycteris* bat CoV HKU4 (NC009019); BatCoV HKU5, *Pipistrellus* bat CoV HKU5 (NC_009020); HCoV-EMC, human betacoronavirus 2c EMC/2012 (JX869059); England1_CoV, human betacoronavirus England 1 (NC_019843); SARS_BatCoV HKU3, severe acute respiratory syndrome (SARS)–related *Rhinolophus* bat CoV HKU3 (DQ022305); SARSr-CoV-CFB, SARS-related Chinese ferret badger CoV (AY545919); SARSr-ciCoV, SARS-related palm civet CoV (AY304488); SARS_huSARS-CoV, SARS human CoV (NC_004718); IBV_Partridge, avian infectious bronchitis virus partridge (AY646283.1); IBV_Peafowl, avian infectious bronchitis virus isolate Peafowl (AY641576); IBV_Beaudette, infectious bronchitis virus strain Beaudette CK (AJ311317); WiCoV HKU20, wigeon CoV HKU20 (NC_016995); NHCoV HKU19, night heron CoV HKU19 (NC_016994); ThCoV HKU12, thrush CoV HKU12 (FJ376621); PorCoV HKU15, porcine CoV HKU15 (NC_016990); MRCoV HKU18, magpie robin CoV HKU18 (NC_016993).

All bat guano samples screened by PCR were negative for NiV and *Histoplasma* spp. but were positive for group C betacoronavirus. The natural reservoir and complete geographic distribution of this CoV are currently unknown. Although we did not isolate live virus from these samples, the detection of nucleic acid and previous isolation of viruses from bat feces and urine (*10*) warrants some concern that guano miners might be exposed to bat pathogens in fresh excreta as well as in soil substances. We suggest that guano miners use preventive measures of personal hygiene and improved barrier protection to reduce the possibility of exposure to zoonotic pathogens.
